# Commentary: No statistically significant difference in long term scarring outcomes of pediatric burns patients treated surgically vs. those treated conservatively

**DOI:** 10.3389/fsurg.2024.1360012

**Published:** 2024-02-13

**Authors:** David E. Most

**Affiliations:** School of Education, Colorado State University, Fort Collins, CO, United States

**Keywords:** scarring, pediatric, scientific inference and reasoning, statistical inference abuse, meta-analytic thinking

A Commentary on No statistically significant difference in long term scarring outcomes of pediatric burns patients treated surgically vs. those treated conservatively By Mistry R, Issa F (2022). Front. Surg. 9:727983. doi:10.3389/fsurg.2022.727983

## Introduction

This commentary aims to provide a constructive critique of the findings of this important and interesting study. As described, this study determined, in a cohort of pediatric burn patients, whether long-term scarring outcomes are different in those who had surgical treatment vs. those who were treated conservatively (1). The Brisbane Burn Scar Impact Profile (BBSIP) was used to measure scarring outcomes. Mean scores for each of the BBSIP questions were reported for both groups, and the scores were compared between groups. In the penultimate paragraph of their manuscript, the authors wrote that “no difference was found in the long-term scar outcomes.” The problem with this claim is that, for many of the BBSIP outcomes, the evidence does not unequivocally support such a conclusion.

## Evidence and interpretation

What does the evidence seem to indicate? Table 3 presents the means and standard deviations, by treatment group, for the responses to each of the 57 questions of the BBSIP. For 31 of the 57 questions, the mean response was identical for both groups with no within-group variability. For those questions, all respondents in both groups selected the lowest possible category regarding impact (“not at all”). However, for the other 26 questions, there are differences between treatment groups, both in terms of central tendency and variability. Although the authors note that part 7 of the BBSIP had the most variation, which focuses on physical symptoms, there seems to be at least as much variability in questions from part 1 regarding the impact on the life of a child. For example, for the fourth question regarding scar treatments, the mean for the surgical treatment group is 1.46, which is ∼0.4 scale points higher than the mean for the conservative treatment group. In addition, the standard deviation for the surgical treatment group is 1.2, which is almost 1 point more than that for the conservative treatment group. Because an individual score cannot be <1, these results indicate that some respondents in the surgical treatment group indicated “somewhat,” the middle of the five possible ordered response categories, for the level of impact. The distribution of responses for this question and, therefore, the observed impact of treatment modality is *not* identical between groups.

Why is there a discrepancy between the evidence offered in Table 3 and the prose characterization of the results? The reason for the discrepancy is a common error in interpretation. The mistake is to conflate a binary statistical declaration with a substantive conclusion. In particular, a declaration of no statistically significant difference is conflated with a scientific conclusion that no evidence was found for a difference or simply of “no difference.” The interpretation of the results, as presented, is based entirely on a binary declaration regarding statistical significance or, equivalently, whether or not a 95% confidence interval (CI) for the difference includes zero, rather than on a substantive evaluation of the magnitude of the difference in means between groups. The difference in standard deviations between groups, which is also a potentially interesting finding, is also not addressed. In short, it is inappropriate to conclude that there is no difference between treatment groups because of a binary statistical decision (e.g., *p*-value > 05, 95% CI for the difference includes zero) (2). On a related note, the group sample sizes presented in Table 3 are a bit misleading as the information presented in the table, including the *p*-values and CIs, is based on the 34 participants who responded to the BBSIP (13 in the surgical group and 21 in the conservative group), rather than the 107 participants from whom responses were solicited. This matters both for proper description and substantive interpretation.

What about uncertainty in the estimates of the difference between groups? The presentation of 95% CIs in Table 3 is helpful for quantifying uncertainty in group differences, although the only interpretation offered by the authors is that all the CIs spanned across zero. Considering, again, the fourth question regarding scar treatments, the plausible true values of the difference in the mean scores between groups, ranges from a high of almost one point on the scale to something close to zero. In other words, if uncertainty is taken into account, while it is plausible that the true difference between the groups is zero, the ranges of plausible true differences in group means that are compatible with the data include many values, up to almost a full category/point on the response scale, which might be considered clinically significant. Embracing this uncertainty further supports the notion that the evidence offered here is not consistent with an interpretation that no difference was found in long-term scar outcomes as a function of treatment.

## Discussion

The concern described here might be considered an example of a more general century-old problem of not distinguishing between statistical inference and scientific inference (3). Empirical examinations of the literature in various disciplines suggest that associated interpretational errors happen more often than not (2). The interpretation offered by the authors in their abstract, that clinicians need not fear the longer-term impact a scar may have when selecting a type of treatment, depends on mistakenly conflating the notion of “no statistically significant difference,” emphasized in the title of the manuscript, with a clinical judgment of no actual differences in outcomes. Instead of focusing on whether or not the true difference between groups could be zero, a better way to make meaning of these data might be to offer a substantive interpretation of the observed differences in the distributions of responses, which brings clinical expertise to bear and that fully embraces statistical and scientific uncertainty. Both generating cumulative knowledge and optimizing clinical outcomes depend on summaries of findings that have fidelity to the evidence.
